# Supplementary material for: Content-rich biological network constructed by mining PubMed abstracts
Source: BMC Bioinformatics. 2004 Oct 8;5:147. doi: 10.1186/1471-2105-5-147 (PMC528731; doi:10.1186/1471-2105-5-147)
Supplement: Additional File 2 — The original results of the above study (non-essential files are deleted to keep the file size under the limit set by BMC bioinformatics). [file 1471-2105-5-147-S2.bz2 › chilibotAdditionalFile2/dip50/606ID10207005E10988/html/LIFR_CNTF.html]

 


 **LIFR** and **CNTF** 
  
Found 72 abstracts in PubMed, retrieved 50.  
 

 What does Google say? 
 PDF only 
| .edu only 

---

**Interactive relationship** (e.g. stimulation, inhibition, etc)

**Inhibitory relationship**- The CDFs leukemia inhibitory factor and ciliary neurotrophic factor  [ **CNTF** ]  bind to a  **LIFR**  gp130 receptor complex to activate Jak signal transducers and activators of transcription and Ras mitogen activated protein kinases signaling pathways.  Ref: 12609984 J Biol Chem< MedlineTA>J Biol Chem,
- The functional receptor complex of  **CNTF**  is composed of the  **CNTF**  receptor alpha CNTFR, gp130 and the leukemia inhibitory factor receptor  [ **LIFR** ]   **LIFR** .  Ref: 11943154 FEBS Lett, 2002
- **CNTF**  functional responses are mediated through a tripartite membrane receptor composed of two signalling receptor chains, gp130 and the leukaemia inhibitory factor receptor  **LIFR** , associated with a non signalling  **CNTF**  binding receptor alpha component CNTFR.  Ref: 11285233 EMBO J, 2001
- Ciliary neurotrophic factor  [ **CNTF** ]  may activate mature astrocytes via binding with the leukemia inhibitory factor receptor  [ **LIFR** ] .  Ref: 11178874 Mol Cell Neurosci, 2001
- Other cytokines oncostatin M OM, leukemia inhibitory factor LIF or ciliary neurotrophic factor  [ **CNTF** ]   **CNTF**  also use the gp130 transducer and induce its heterodimerization with LIF receptor  **LIFR** .  Ref: 9110148 Eur Cytokine Netw, 1997
- The functional receptor complex of ciliary neurotrophic factor  [ **CNTF** ]   **CNTF** , a member of the gp130 family of cytokines, is composed of  **CNTF** , the  **CNTF**  receptor alpha CNTFR, gp130 and the leukemia inhibitory factor receptor  [ **LIFR** ]   **LIFR** .  Ref: 12707266 J Biol Chem< MedlineTA>J Biol Chem, 1997
- Its receptor complex consists of a ligand binding component,  **CNTF**  receptor, associated with two signaling receptor components, gp130 and leukemia inhibitory factor receptor  [ **LIFR** ] .  Ref: 11294841 J Biol Chem, 2001
- For signal transduction,  **CNTF**  requires a receptor complex, composed of the IL 6 signal transducing molecule gp130, leukemia inhibitory factor receptor  [ **LIFR** ]   **LIFR**  beta, and CNTFR alpha.  Ref: 11464862 Med Pediatr Oncol, 2001
- The low affinity leukaemia inhibitory factor receptor LIF R  [ **LIFR** ]  is a component of cell surface receptor complexes for the multifunctional cytokines leukaemia inhibitory factor, ciliary neurotrophic factor  [ **CNTF** ] , oncostatin M and cardiotrophin 1.  Ref: 9396734 Biochem J, 1997
- The leukemia inhibitory factor receptor  [ **LIFR** ]  LIF R  [ **LIFR** ]  subunit is a component of cell surface receptor complexes for the multifunctional cytokines, LIF, cardiotrophin 1, ciliary neurotrophic factor  [ **CNTF** ] , and human oncostatin M.  Ref: 11042511 Leuk Lymphoma, 2000
- Ciliary neurotrophic factor  [ **CNTF** ]   **CNTF**  drives the sequential assembly of a receptor complex containing the ligand specific alpha receptor subunit CNTFR alpha and the signal transducers gp130 and leukemia inhibitory factor receptor  [ **LIFR** ]  beta  **LIFR** .  Ref: 8799186 Proc Natl Acad Sci U S A, 1996
- Ciliary neurotrophic factor  [ **CNTF** ]   **CNTF**  associates with an alpha subunit CNTFRalpha of the receptor complex to initiate signal transduction by facilitating heterodimerization of the gp130 transducing protein and the leukemia inhibitory factor receptor  [ **LIFR** ]   **LIFR**  beta.  Ref: 8824245 J Biol Chem, 1996
- Ciliary neurotrophic factor  [ **CNTF** ]   **CNTF**  drives the sequential assembly of a receptor complex containing the ligand specific alpha receptor subunit CNTFR and the signal transducing beta subunits gp130 and leukemia inhibitory factor receptor  [ **LIFR** ]  beta  **LIFR** .  Ref: 9287306 J Biol Chem, 1997
- The heterodimeric cytokine composed of the soluble ciliary neurotrophic factor  [ **CNTF** ]  receptor sCNTFR and the IL 6 family member cardiotrophin like cytokine CLC was recently identified as a new ligand for gp130 leukemia inhibitory factor receptor  [ **LIFR** ]   **LIFR**  complex Plun Favreau, H.  Ref: 11952795 Eur J Biochem, 2002
**Neutral relationship**- **CNTF**  induced the tyrosine phosphorylation of  **LIFR**  and gp130, as well as of proteins with the molecular weights of 88 91 and 42 kDa.  Ref: 10082809 Brain Res, 1999
- These data indicate that although LIF and Hyper  **CNTF**  use the same heterodimeric receptor complex of gp130 and  **LIFR** , only Hyper  **CNTF**  induces neuronal differentiation.  Ref: 12071967 Eur J Biochem, 2002
- Here, we demonstrate that human  **CNTF**  cannot directly induce a heterodimer of human gp130 and  **LIFR** .  Ref: 12643274 J Biol Chem, 2003
- Here, we demonstrate that human  **CNTF**  cannot directly induce a heterodimer of human gp130 and  **LIFR** .  Ref: 12517918 J Biol Chem< MedlineTA>J Biol Chem, 2003
- The resulting IL 6  **CNTF**  chimera lost the capacity to signal via gp130 on cells without  **LIFR** , but acquired the ability to signal via the gp130  **LIFR**  heterodimer and STAT3 on responsive cells.  Ref: 10207005 J Biol Chem, 1999
- Signal transduction by  **CNTF**  requires that it bind first to  **CNTF**  R, permitting the recruitment of gp130 and LIF R  [ **LIFR** ] , forming a tripartite receptor complex.  Ref: 12765290 J Zhejiang Univ Sci< MedlineTA>J Zhejiang Univ Sci, 1999
- These data suggest that both the gp130 CBD and the  **LIFR**  CBD1 interact with CNTFR in a similar way and provide insights into the nature of the  **CNTF**  receptor complex.  Ref: 12707266 J Biol Chem< MedlineTA>J Biol Chem, 1999
- Hyper  **CNTF**  efficiently induced dose dependent STAT3 phosphorylation and proliferation of BAF 3 cells stably transfected with gp130 and LIF R  [ **LIFR** ]  cDNAs.  Ref: 12071967 Eur J Biochem, 2002
- The D1 structural motif, located at the beginning of the D helix of human  **CNTF** , contains two amino acid residues, F152 and K155, which are conserved among all cytokines that signal through  **LIFR** .  Ref: 8799186 Proc Natl Acad Sci U S A, 1996
- The functional receptor for LIF is composed of  **LIFR**  alpha chain LIFRalpha and gp130 both of which are shared in the functional receptors for oncostatin M, ciliary neurotrophic factor  [ **CNTF** ] , and cardiotrophin 1.  Ref: 9852103 J Biol Chem, 1998
- Cells that only express gp130 and LIF R  [ **LIFR** ] , but not  **CNTF**  R are refractory to stimulation by  **CNTF** .  Ref: 12765290 J Zhejiang Univ Sci< MedlineTA>J Zhejiang Univ Sci, 1998
- An antagonist for the leukemia inhibitory factor receptor  [ **LIFR** ]  inhibits leukemia inhibitory factor, cardiotrophin 1, ciliary neurotrophic factor  [ **CNTF** ] , and oncostatin M.  Ref: 9341130 J Biol Chem, 1997

**Non-interactive relationship** (e.g. studied together, co-existance, homology, etc.)

- While on BAF3 gp130 LIF R  [ **LIFR** ]  cells, Hyper  **CNTF**  and LIF exhibited similar biological responses, the activity of Hyper  **CNTF**  on pheochromocytoma cells PC12 cells was quite distinct from that of LIF.  Ref: 12071967 Eur J Biochem, 2002
- The mRNA levels of neuropoietic cytokines, ciliary neurotrophic factor  [ **CNTF** ]   **CNTF** , leukemia inhibitory factor LIF, and interleukin 6 IL 6, and their receptor components CNTFR alpha,  **LIFR**  beta, IL 6R alpha, and gp130 were examined in seventy six patients with various peripheral neuropathies to determine the extent of expression of these cytokines and receptors, and their relationship to nerve fiber pathology and cell infiltration in the diseased nerves.  Ref: 11358282 Neurochem Res, 2001
- The interleukin 6 IL 6 family of cytokines activates signaling through the formation of either gp130 homodimers, as for IL 6, or gp130 leukemia inhibitory factor receptor  [ **LIFR** ]   **LIFR**  heterodimers as for ciliary neurotrophic factor  [ **CNTF** ]   **CNTF** , leukemia inhibitory factor, oncostatinM, and cardiotrophin 1.  Ref: 8940087 J Biol Chem, 1996
- gp130 either homodimerizes in response to IL 6 and IL 11 or forms heterodimers with the leukemia inhibitory factor LIF receptor  **LIFR**  in response to LIF, oncostatin M OSM, ciliary neurotrophic factor  [ **CNTF** ]   **CNTF** , cardiotrophin 1 CT 1 or cardiotrophin like cytokine resulting in the onset of cytoplasmic tyrosine phosphorylation cascades.  Ref: 12047380 Eur J Biochem, 2002
- In order to investigate the effects of  **CNTF**  on fat cells, we examined the expression of  **CNTF**  receptor complex proteins  **LIFR** , gp130, and CNTFRalpha during adipocyte differentiation and the effects of  **CNTF**  on STAT, Akt, and MAPK activation.  Ref: 12424252 J Biol Chem, 2003
- In addition, the expression of ciliary neurotrophic factor  [ **CNTF** ]  receptor CNTFR and leukemia inhibitory factor receptor  [ **LIFR** ]   **LIFR**  mRNAs, but not of interleukin 6 receptor mRNA, was up regulated after systemic lipopolysaccharide treatment.  Ref: 12127095 Neuroscience, 2002
- In this study, we provide the first demonstration that the membrane distal CBD CBD1 of  **LIFR**  associates in vitro with soluble CNTFR in the absence of  **CNTF** .  Ref: 11943154 FEBS Lett, 2002
- Eighteen pituitary adenoma biopsies removed in transsphenoidal surgery six corticotrophic adenomas, four nonfunctioning adenomas, four somatotrophinomas, four prolactinomas and six normal anterior pituitaries were examined for the expression of IL 6 receptor R, leukemia inhibitory factor LIF, LIF R  [ **LIFR** ] , IL 11, IL 11 R, oncostatin M OSM, OSM R, ciliary neurotrophic factor  [ **CNTF** ]   **CNTF** ,  **CNTF**  R and cardiotrophin 1 CT 1.  Ref: 11095488 J Clin Endocrinol Metab, 2000
- Novel neurotrophin 1 B cell stimulating factor 3 is a recently cloned gp130 cytokine, acting through the tripartite ciliary neurotrophic factor  [ **CNTF** ]  receptor alpha leukemia inhibitory factor receptor  [ **LIFR** ]  gp130 receptor complex.  Ref: 12639901 Endocrinology, 2003
- Analyses of the activation level of leukemia inhibitory factor receptor  [ **LIFR** ]  beta and signal transducer and activator of transcription 3 signaling molecules in response to exogenous  **CNTF**  revealed an increased tyrosine phosphorylation positively correlated with the twitch tension developed by the soleus muscle of the animals.  Ref: 9952403 J Neurosci, 1999
- We concluded that the fusion protein Hyper  **CNTF**  had superagonistic activity on target cells expressing gp130 and LIF R  [ **LIFR** ] , but lacking membrane bound  **CNTF**  R.  Ref: 12765290 J Zhejiang Univ Sci< MedlineTA>J Zhejiang Univ Sci, 1999
- Human  **CNTF**  elicits cellular responses by induction of a receptor complex consisting of the  **CNTF**  alpha receptor CNTFR, which is not involved in signal transduction, and the beta receptors gp130 and leukemia inhibitory factor receptor  [ **LIFR** ]   **LIFR** .  Ref: 12643274 J Biol Chem, 2003
- Besides identifying a specific  **LIFR**  binding epitope on  **CNTF** , our results suggest that receptor recognition sites of cytokines are organized as modules that are exchangeable even between cytokines with limited sequence homology.  Ref: 10207005 J Biol Chem, 1999
- Using molecular modeling we defined a putative  **LIFR**  binding epitope on  **CNTF**  that consists of three distinct regions C terminal A helix N terminal AB loop, BC loop, C terminal CD loop N terminal D helix.  Ref: 10207005 J Biol Chem, 1999
- Our studies clearly demonstrate that the expression of two of the three  **CNTF**  receptor complex components, CNTFRalpha and  **LIFR** , decreases during adipocyte differentiation.  Ref: 12424252 J Biol Chem, 2003
- Identification of ciliary neurotrophic factor  [ **CNTF** ]   **CNTF**  residues essential for leukemia inhibitory factor receptor  [ **LIFR** ]  binding and generation of  **CNTF**  receptor antagonists.  Ref: 8799186 Proc Natl Acad Sci U S A, 1996
- ... at high concentrations, it can directly induce a signaling heterodimer of human gp130 and human  **LIFR**  in the absence of the  **CNTF**  receptor.  Ref: 12643274 J Biol Chem, 2003
- Transcripts of the remaining two components of the  **CNTF**  receptor system, gp130 and  **LIFR**  beta, were found by Northern blotting in 83% and 70% of the tumors, respectively.  Ref: 10720204 J Neurooncol, 1999
- The proliferative results confirmed that  **CNTF**  required homodimerization of the gp130,  **CNTF**  R and LIF R  [ **LIFR** ]  receptor subunit whereas Hyper  **CNTF**  required heterodimerization of the gp130 and LIF R  [ **LIFR** ]  receptor subunit.  Ref: 12765290 J Zhejiang Univ Sci< MedlineTA>J Zhejiang Univ Sci, 1999
- ...  **CNTF**  and leukemia inhibitory factor LIF signal via a heterodimer of gp130 and LIF receptor  **LIFR** .  Ref: 10207005 J Biol Chem, 1999
- Using RT PCR, expression of ciliary neurotrophic factor  [ **CNTF** ]  receptor alpha, leukemia inhibitory factor receptor  [ **LIFR** ] , and gp130 could be demonstrated in mRNA derived from murine corticotroph AtT 20 cells and murine pituitary tissue.  Ref: 12639901 Endocrinology, 2003
- ... at high concentrations, it can directly induce a signalling heterodimer of human gp130 and human  **LIFR**  in the absence of the  **CNTF**  receptor.  Ref: 12517918 J Biol Chem< MedlineTA>J Biol Chem, 2003
- Semiquantitative reverse transcription polymerase chain reaction RT PCR analysis showed that IL 11 and its receptor,  **CNTF**  and its receptor,  **LIFR** , and gp130 were constitutively expressed throughout the culture period.  Ref: 12110437 Bone, 2002
- Expression of mRNAs for ciliary neurotrophic factor  [ **CNTF** ]   **CNTF** , leukemia inhibitory factor LIF, interleukin 6 IL 6, and their receptors CNTFR alpha,  **LIFR**  beta, IL 6R alpha, and gp130 in human peripheral neuropathies.  Ref: 11358282 Neurochem Res, 2001
- Signal transduction by  **CNTF**  requires that it bind first to CNTFR alpha, permitting the recruitment of gp130 and  **LIFR**  beta, forming a tripartite receptor complex.  Ref: 10812968 Pharm Acta Helv, 2000
- Human  **CNTF**  elicits cellular responses by induction of a receptor complex consisting of the  **CNTF**  a receptor CNTFR, which is not involved in signal transduction, and the b receptors gp130 and leukaemia inhibitory factor receptor  **LIFR** .  Ref: 12517918 J Biol Chem< MedlineTA>J Biol Chem, 2000
- A convergent set of results supports the hypothesis that the  **CNTF**  effect is due to the illegitimate binding on the leukemia inhibitory factor receptor  [ **LIFR** ]   **LIFR**  i it requires high concentration of recombinant rat  **CNTF** .  Ref: 11178874 Mol Cell Neurosci, 2001
- On these bases, we propose a mechanistic model in which, in the adult brain, a  **CNTF**   **LIFR**  interglial system may be modulated by neurons that synthesize CNTFRalpha.  Ref: 11178874 Mol Cell Neurosci, 2001
- These results suggest that TrkB, a functional high affinity receptor of BDNF and NT 4 5, and  **LIFR**  beta, a receptor component contained in  **CNTF**  and LIF receptor complex, might be involved in the observed synergistic effects.  Ref: 10366699 Brain Res Dev Brain Res, 1999
- Transfer of the leukemia inhibitory factor receptor  [ **LIFR** ]  binding site from ciliary neurotrophic factor  [ **CNTF** ]  to interleukin 6.  Ref: 10207005 J Biol Chem, 1999
- Differential temporal expression of mRNAs for ciliary neurotrophic factor  [ **CNTF** ]   **CNTF** , leukemia inhibitory factor LIF, interleukin 6 IL 6, and their receptors CNTFR alpha,  **LIFR**  beta, IL 6R alpha and gp130 in injured peripheral nerves.  Ref: 9630704 Brain Res, 1998
- Here, we report the construction and expression of a  **CNTF**  soluble  **CNTF**  receptor sCNTF R fusion protein Hyper  **CNTF**  with enhanced biological activity on cells expressing gp130 and leukemia inhibitory factor receptor  [ **LIFR** ]  LIF R  [ **LIFR** ] , but not membrane bound  **CNTF**  R.  Ref: 12071967 Eur J Biochem, 2002
- We previously reported that the membrane distal cytokine binding domain CBD1 of  **LIFR**  could interact in vitro with soluble CNTFR sCNTFR in the absence of  **CNTF** .  Ref: 12707266 J Biol Chem< MedlineTA>J Biol Chem, 2002
- The expression of all other cytokines LIF, IL 11,  **CNTF**  and receptors IL 6 R, LIF R  [ **LIFR** ] , IL 11 R, OSM R,  **CNTF**  R was found in different patterns in the adenoma subtypes and normal pituitaries.  Ref: 11095488 J Clin Endocrinol Metab, 2000
- Two new types of  **CNTF**  variants, characterized by weakened interactions with either CNTFR or both  **LIFR**  and gp130, were developed, and the biological activities of these and other mutants were determined in non neuronal versus neuronal cells, as well as in non neuronal cells transfected with an expression vector for CNTFR.  Ref: 9287306 J Biol Chem, 1997
- Leukemia inhibitory factor LIF, cardiotrophin 1, ciliary neurotrophic factor  [ **CNTF** ] , and oncostatin M OSM lead to heterodimerization of LIF receptor  **LIFR**  or the OSM specific receptor OSMR with glycoprotein gp 130, the common receptor subunit for IL 6 type cytokines.  Ref: 10586060 J Immunol, 1999
- Given the restricted expression domain of the  **CNTF**  receptor alpha CNTFR in the developing forebrain germinal zone and adult forebrain periventricular area, we have examined the putative role of CNTFR  **LIFR**  gp130 mediated signaling in regulating forebrain neural stem cell fate in vivo and in vitro.  Ref: 11567054 J Neurosci, 2001
- Continuous treatment with Dex 10 8 mol L inhibited the endogenous production of IL 6, LIF, OSM, IL 11R, and OSMR, but had no detectable effect on the expression of IL 11, CT 1,  **CNTF** , CNTFR,  **LIFR** , or gp130.  Ref: 12110437 Bone, 2002
- The leukemia inhibitory factor receptor  [ **LIFR** ]  LIF R  [ **LIFR** ]  is activated not only by LIF, but also by cardiotrophin 1, ciliary neurotrophic factor  [ **CNTF** ]  with its receptor, and oncostatin M OSM.  Ref: 9341130 J Biol Chem, 1997
